# P1 adhesin genotype characteristics of *Mycoplasma pneumoniae* in China from 2017 to 2019

**DOI:** 10.3389/fcimb.2025.1513177

**Published:** 2025-02-17

**Authors:** Shirong Li, Haiwei Dou, Dawei Shi, Ruijie Yuan, Peng Tu, Qing Yuan, Deli Xin, Wenjie Qi

**Affiliations:** ^1^ Department of Infectious Disease, Beijing Friendship Hospital, Capital Medical University, Beijing, China; ^2^ Department of Respiratory, Beijing Fengtai Hospital of Traditional Chinese Medicine (Nan Yuan Hospital), Beijing, China; ^3^ Department of Pediatrics, Beijing Friendship Hospital, Capital Medical University, Beijing, China; ^4^ Department of Geriatric Medicine, Henan Provincial People’s Hospital, People’s Hospital of Zhengzhou University, People’s Hospital of Henan University, Zhengzhou, Henan, China; ^5^ Department of Pediatrics, Traditional Chinese and Western Medicine Hospital of Wuhan, Tongji Medical College, Huazhong University of Science and Technology; Wuhan No.1 Hospital, Wuhan, China; ^6^ Department of Respiratory, Branch of Dongzhimen Hospital Affiliated to Beijing University of Chinese Medicine, Luoyang Hospital of TCM, Henan, China

**Keywords:** *Mycoplasma pneumoniae*, genotype, P1 gene, community-acquired pneumonia, prevalence

## Abstract

**Introduction:**

*Mycoplasma pneumoniae* is one of the important pathogens of community-acquired pneumonia (CAP), and P1 adhesin serves as a pathogenic protein and an immune protein involved in the pathogenesis of *mycoplasma pneumoniae*. The aim of this study was to investigate the P1 adhesin genotype in *Mycoplasma pneumoniae* and its association with disease severity in patients with CAP from 2017 to 2019.

**Methods:**

*M. pneumoniae* was identified in patient samples by real-time quantitative polymerase chain reaction (qPCR). The P1 genotypes of samples were determined using a culture-independent P1 typing method.

**Results:**

In total, 1,907 clinical samples were collected from 13 tertiary hospitals in Beijing, Shenyang, and Baotou, including 1488 samples from children and 419 from adults. Of these, 820 samples (43.00%), including 777 from children and 43 from adults, were positive for *M. pneumoniae*. 797 samples were successfully typed using the culture-independent P1 typing method (P1-1, 605; P1- 2, 192). The *M. pneumoniae* detection rate and P1-1 detection rate differed significantly between children and adults (both *p* < 0.01), with P1-1 remaining the dominant genotype. The proportion of P1-2 samples increased in children from 16.75% in 2017 to 28.76% in 2019.

**Discussion:**

No relationship between the P1 genotype and disease severity was identified. Monitoring the genotype changes of P1 adhesin in local populations may positively impact the epidemiological prevention and control of *M. pneumoniae* infections.

## 1 Introduction

*Mycoplasma pneumoniae* is the most prevalent cause of community-acquired pneumonia (CAP) in children, especially among hospitalized patients (Morozumi et al., 2008; Jain et al., 2015; Meyer Sauteur et al., 2020b; Meyer Sauteur et al., 2020a). *M. pneumoniae* regularly causes epidemics every 3–7 years that last for 1–2 years (Dumke et al., 2015). The P1 adhesin, located at the tip of the *M. pneumoniae* attachment organelle (Kenri et al., 2019), is an essential determinant for adherence to respiratory tract epithelial cells and for sliding movement (Krause and Baseman, 1982). Together with the P40/90 polypeptide, it forms a cross-membrane complex known as “NAP” (Scheffer et al., 2017). Additionally, P1 adhesin is recognized as one of the immunodominant proteins and performs important functions in the pathogenesis of *M. pneumoniae* infection (Peng et al., 2023) and immune response of infected patients (Vizarraga et al., 2020). *M. pneumoniae* can be divided into P1 types 1 (P1-1) and 2 (P1-2) based on differences of two repetitive DNA regions (RepMP2/3 and RepMP4) in the P1 protein-coding gene (Dumke et al., 2004; Dumke et al., 2006). Studies on the P1 genotype and its variation have been conducted worldwide (Sasaki et al., 1996; Kenri et al., 2008; Jacobs et al., 2015; Jiang et al., 2021; Guo et al., 2022; Wang et al., 2022), and changes in P1 genotype are believed to be associated with the prevalence of *M. pneumoniae* infections (Atkinson et al., 2008). However, findings regarding the relation between the P1 genotype and disease severity have been inconsistent. The culture-independent P1 genotype method for *M. pneumoniae* has been widely used due to its efficiency, cost-effectiveness, and ability to directly classify clinical samples without relying on mycoplasma culture (Dumke et al., 2006). An accurate understanding of the P1 genotype and its variation in China, as well as the correlation between genotype and disease severity, is essential for disease management and improving early warning systems.

Thus, this study investigated the changes of the P1 genotype in patients infected with *M. pneumoniae* in China from 2017 to 2019 and the relationship of the genotype with disease severity.

## 2 Materials and methods

### 2.1 Ethics approval

This study was approved by the Medical Ethics Committee of Beijing Friendship Hospital, Capital Medical University (2019-P2-176-02). Written authorization was obtained from the patients or their guardians (for pediatric patients) prior to sample collection.

### 2.2 Patients and clinical samples

We followed the guidelines for diagnosis and treatment of CAP in children (Revised WHO Classification and Treatment of Pneumonia in Children at Health Facilities: Evidence Summaries, 2014; Haq et al., 2017) and adults (Metlay et al., 2019; Olson and Davis, 2020) to diagnose CAP and assess its severity. All patients were consistent with community-associated infection, characterized by new or worsening symptoms such as cough, sputum, fever, and other respiratory signs, along with evidence of lung consolidation or wet rales, and imaging-suggested inflammation. Children showing hypoxemia, central cyanosis, severe respiratory distress, refusal to feed, dehydration, or disturbances in consciousness (e.g., drowsiness, coma, convulsions) were considered to have severe pneumonia. The IDSA/ATS CAP severity criteria were applied to assess the severity of pneumonia in adults (Metlay et al., 2019). Pharyngeal swab samples were collected from patients with CAP and no chronic diseases, immune system diseases, or inherited metabolic diseases in Beijing, Shenyang, and Baotou. The samples in Beijing were collected at 11 tertiary hospitals, including Beijing Friendship Hospital, Capital Medical University; Peking University First Hospital; Beijing Children’s Hospital, Capital Medical University; Peking University Third Hospital; Beijing Chao-Yang Hospital, Capital Medical University; Civil Aviation General Hospital; Beijing Chang Ping District Hospital of Integrated Traditional Chinese and Western Medicine; Emergency General Hospital; Dongfang Hospital, Beijing University of Chinese Medicine; The First Hospital of Tsinghua University; and China-Japan Friendship Hospital. In Shenyang, the samples were collected from Sheng Jing Hospital of China Medical University, and the samples in Baotou were collected from The Fourth Hospital of Baotou (Baotou Children’s Hospital).

For each sample, the following information was recorded: the age and sex of the patient, the sampling year, the sampling city, the treatment status of the patient (inpatient or outpatient), and the presence of severe or general pneumonia (**Table 1**). Samples were collected according to specifications and stored in PPLO medium (one liter contains 0.4 g of PPLO broth powder [255420, Becton, Dickinson and Company, Frankin Lakes, NJ, USA], 0.53 g of polypeptone powder [LP0042, Japan Pharmaceutical Co., Yokohama, Japan], 1 g of tryptone powder [394-00115, Oxoid, Thermo Fisher Scientific, Waltham, MA, USA], and 0.04 g of herring protamine powder [D3159, Sigma-Aldrich, St. Louis, MO, USA] sterilized *via* autoclaving, as well as 3.5 mL of 25% yeast extract, 10 mL of 2% yeast extract, 17 mL of fetal bovine serum [F2442, Sigma-Aldrich), 2 mL of 50% glucose for injection, 0.8 mL of phenol red indicator, and 1 mmol sodium hydroxide solution [0.6 mL/100 mL]). After mixing, the sample medium was evenly aliquoted into three 1.5 ml EP tubes: one for DNA extraction, one for strain culture, and one for future use. All samples were transported in dry ice and stored at −80°C until use. Only one sample (throat swab) from each patient was included in the study.

**Table 1:** Patient demographics.

Year	City	Source	Outpatients	Inpatients Total (Severe)	P1-1	P1-2	Total
**2017**	Beijing*	Pediatric	262	163 (40)	124	24	425
		Adults	0	419 (0)	21	16	419
	Shenyang**	Pediatric	0	122 (49)	40	9	122
**2018**	Beijing*	Pediatric	137	320 (103)	207	57	457
**2019**	Beijing*	Pediatric	94	346 (267)	183	79	440
	Baotou	Pediatric	0	44 (36)	30	7	44
**Total**	493			1414 (495)			1907

*Samples were collected from 11 hospitals in Beijing. ** Samples were collected only in one hospital.

### 2.3 *M. pneumoniae* strains

The *M. pneumoniae* reference strains ATCC 29342 (M129 strain) and ATCC 15531 (FH strain) were used as positive controls.

### 2.4 Quantitative polymerase chain reaction (qPCR)

Total DNA was extracted from *M. pneumoniae* using a bacterial genomic DNA kit (CoWin Biosciences, Jiangsu, China) according to the manufacturer’s instructions. PCR was performed in a total volume of 20 μL reaction containing 0.3 μL each of forward and reverse primers(1μmol/L), 10 μL of 2× buffer UltraSYBR Mixture (CoWin Biosciences), 2 μL of genomic DNA, and 7.4 μL of ddH_2_O. The PCR reaction was initiated at 95°C for 2 min, followed by 40 cycles of denaturing at 95°C for 10 seconds (s), and annealing at 55°C for 30 s, then and elongation at 72°C for 60 s. A melting curve analysis was conducted by heating the PCR products at 95°C for 15 s, colling down to 550°C for 30 s, and then 15 s each at 0.5°C increments between 55°C and 95°C. The PCR products were further confirmed by sequencing using the service of Shanghai Sangon Bioengineering Co., Ltd. (Shanghai, China).

The following primers for PCR were synthesized by Shanghai Sangon Bioengineering Co., Ltd. (Shanghai, China): 23S-F, GACACCCGTTAGGCGCAA; and 23S-R, CTGGATAACAGTTACCAATTAGAACAGC.

### 2.5 Culture-independent P1 typing of *M. pneumoniae*

Culture-independent P1 typing of *M. pneumoniae* was performed using duplex PCR, as described by Zhao et al (Zhao et al., 2015). PCR primers Type1-F and Type1-R, and the Type1-P probe were used to detect P1-1 DNA in samples, whereas the primers Type2-F and Type2-R, and the Type2-P probe were used to detect P1-2 DNA. The amplification conditions were as follows: 95°C for 10 min, followed by 45 cycles of 95°C for 15 s and 56°C for 15 s.

The sequences of primers for P1 genotyping, which were synthesized by Eurogentec (Seraing, Belgium), were as follows: Type1-F, CCAGATTCACGTTTAATTTC; Type1-R, GCATCTAACATGAAGACTG; Type1-P, 5′6-FAM-AACCAACAACTTCTCATTCATCCTCAG-3′BHQ1; Type2-F, TTGGGTAAACCTAATTTGC; Type2-R, ACACGTATTAGCATCACTA; and Type2-P, 5′VIC-AAGACTATTCGCCTTACAACCAACC-3′BHQ1.

### 2.6 *M. pneumoniae* culture

When a clinical sample tested positive by PCR, the aliquot designated for strain culture was inoculated into PPLO medium and incubated at 37°C (Liu et al., 2014). The strains were cultured on agar plates for at least 72 hours before other experiments were performed.

### 2.7 Data analysis

All statistical analyses were performed using SPSS 20.0 (IBM, Armonk, NY, USA). Categorical variables were presented as counts and percentages. Continuous variables were presented as the median (interquartile range). The detection rates of *M. pneumoniae* and mutations in each group were analyzed using the chi-squared test. The P1 genotype results of the different methods and groups were analyzed using the chi-squared test or paired chi-squared test. *p* < 0.05 indicated statistical significance.

## 3 Results

### 3.1 Characteristics of the collected samples

In total, we collected 1907 clinical samples between 2017 and 2019, including 1488 samples from pediatric patients in Beijing, Shenyang, and Baotou. Meanwhile, 493 samples were collected from outpatients, and 1414 samples collected from inpatients. Among the inpatients, 495 and 919 were diagnosed with severe pneumonia and general pneumonia (mild to moderate), respectively, as detailed in **Table 1**.

### 3.2 *M. pneumoniae* detection in clinical samples

qPCR revealed positivity for *M. pneumoniae* in 820 clinical samples, including 777 samples from children (positivity rate = 52.22%) and 43 samples from adults (positivity rate = 10.26%, **Table 2**). The rate of *M. pneumoniae* infection was higher in children with CAP than in adults (*p* < 0.01).

**Table 2:** The detection rate of *M. pneumoniae* in clinical samples from patients with CAP.

	Adults	Children	Total	*X^2^*	*P*
***M. pneumoniae* positive**	43 (10.26%)	777 (52.22%)	820 (43.00%)	234.80	< 0.01
***M. pneumoniae* negative**	376 (87.74%)	711 (47.78%)	1,087 (57.00%)		
**Total**	419	1488	1907		

### 3.3 Culture-independent P1 genotype of clinical samples

Of the 820 *M. pneumoniae*-positive clinical samples, 23 failed to be typed, including 17 from children and 6 from adults. Therefore, 760 pediatric and 37 adult samples were submitted for further analysis.

### 3.4 *M. pneumoniae* detection in pediatric patients

#### 3.4.1 *M. pneumoniae* infection and P1 genotype characteristics in pediatric patients

The median age of pediatric patients was 6.82 years (range: 1–16) of the P1 genotype successed, and the group included 355 boys (46.71%) and 405 girls (53.29%). In total, 197 (25.92%), 264 (34.74%), and 299 samples (39.34%) were collected in 2017, 2018, and 2019, respectively. Of these, 109 samples (14.34%) were from outpatients and 651 samples (85.66%) were from inpatients. Among the inpatients, 227 (34.87%) were diagnosed with severe pneumonia, and 424 (65.13%) with general pneumonia (**Table 3**).

**Table 3:** Characteristics of the P1 adhesin genotype of *M. pneumoniae* in clinical samples from pediatric patients with CAP.

Characteristics	Total	P1-1	P1-2	*X^2^*	*P*
**No. of subjects**	760	584 (76.84%)	176 (23.16%)		
**Boys/girls**	355/405	276/308	79/97	0.306	0.58
**Median age (IQR)**	7.00 (5.00-9.00)	7.00 (5.00-9.00)	7.00 (4.25-9.00)	9.155	0.87
Collection city					
Beijing^a, b^	674	514 (76.26%)	160 (23.74%)	1.13	0.57
Shenyang^a^	49	40 (81.63%)	9 (18.37%)		
Baotou^b^	37	30 (81.08%)	7 (18.92%)		
Collection year					
2017	197	164 (83.25%)	33 (16.75%)	10.19	0.01
2018	264	207 (78.41%)	57 (21.59%)		
2019	299	213 (71.24%)	86 (28.76%)		
**Outpatients**	109	86 (78.90%)	23 (21.10%)	0.303	0.58
**Inpatients**	651	498 (76.50%)	153 (23.50%)		
Severe pneumonia	227	167 (73.57%)	60 (26.43%)	1.664	0.20
General pneumonia	424	331 (78.07%)	93 (21.93%)		

IQR, interquartile range. a: The P1 adhesin genotype results for Beijing and Shenyang in 2017 showed no significant difference (X^2^=0.122, *p*=0.727). b; The P1 adhesin genotype for Beijing and Baotou in 2019 showed no significant difference (X^2^=1.997, *p*=0.158).

Sex (*p* = 0.58), age (*p* = 0.87), sample origin (*p* = 0.58), and the inpatient/outpatient status (*p* = 0.58) did not differ between P1-1 and P1-2. In addition, the prevalence of general pneumonia and severe pneumonia did not differ between the genotypes (*p* = 0.20), indicating that the P1 genotype does not determine clinical disease severity in children.

The proportion of P1-2 cases increased from 16.75% in 2017 to 28.76% in 2019, whereas the proportion of P1-1 cases consequently decreased over this period from 83.25% in 2017 to 71.24% in 2019 (**Table 3**). The difference in the rate of P1-2 positivity over the study period was statistically significant (*p* = 0.006). Furthermore, the P1-2 positivity rate was higher in 2019 than in 2017 (*p* = 0.002, χ^2^ = 9.395).

#### 3.4.2 Regional characteristics and P1 genotype trends of *M. pneumoniae* infection in pediatric patients

In 2017, 124 P1-1 samples (83.78%) and 24 P1-2 samples (16.22%) were collected in Beijing, whereas 40 P1-1 samples (81.63%) and 9 P1-2 samples (18.37%) were collected in Shenyang. Further, in 2019, 183 P1-1 samples (69.85%) and 79 P1-2 samples (30.15%) were obtained in Beijing, whereas 30 P1-1 samples (81.08%) and 7 P1-2 samples (18.92%) were collected in Baotou (**Table 4**). These findings indicate that the P1 genotype did not differ between Beijing and Shenyang samples in 2017 or between Beijing and Baotou samples in 2019.

**Table 4:** Culture-independent P1 adhesin genotype of clinical samples from pediatric patients with CAP collected in Beijing and Shenyang (2017) and Beijing and Baotou (2019).

P1 genotype	2017					2019				
	Beijing	Shenyang	Total	X^2^	*P*	Beijing	Baotou	Total	*X^2^*	*P*
**P1-1**	124 (83.78%)	40 (81.63%)	164 (83.25%)	0.12	0.73	183 (69.85%)	30 (81.08%)	213 (71.24%)	1.99	0.16
**P1-2**	24 (16.22%)	9 (18.37%)	33 (16.75%)			79 (30.15%)	7 (18.92%)	86 (27.76%)		
**Total**	148	49	197			262	37	299		

Of the 674 samples collected in Beijing, 514 (76.26%) carried P1-1 strains, and 160 (23.74%) carried P1-2 strains. The proportion of P1-1 strains in children decreased from 83.78% in 2017 to 78.41% in 2018 and 69.85% in 2019, whereas the proportion of P1-2 strains increased from 16.22% in 2017 to 30.15% in 2019.

### 3.5 *M. pneumoniae* infection in adult patients

#### 3.5.1 *M. pneumoniae* infection and P1 genotype characteristics in adult patients

The median age of adult patients was 47.57 years (range: 18–84), and these patients included 10 men (23.26%) and 33 women (76.74%). All samples were collected from inpatients with general pneumonia in Beijing in 2017. Of the genotyped samples, 21 (56.76%) carried the P1-1 genotype, and 16 (43.24%) carried the P1-2 genotype. Patient sex (*p* = 0.49) and age (*p* = 0.35) did not differ between the genotypes (**Table 5**).

**Table 5:** Demographics of adult patient with CAP and *M. pneumoniae* P1 adhesin genotype identified in 2017.

Characteristics	Total	P1-1	P1-2	*X^2^*	*P*
No. of subjects	37	21 (56.76%)	16 (43.24%)	1.35	0.25
Male/Female	9/28	6/15	3/13	0.48	0.49
Median age	47.57 ± 15.25	44.62 ± 13.52	51.44 ± 16.91		0.35

### 3.6 Differences between pediatric and adult patients with *M. pneumoniae* CAP

A significantly lower proportion of adults carried the P1-1 genotype than children in 2017 (56.76% vs. 83.25%, χ^2^ = 13.21, *p* < 0.01, **Tables 5**, **3**). In total, 164 P1-1 (83.25%) and 33P1-2 samples (16.75%) were collected from children, highlighting the significantly higher detection rate of the P1-2 genotype in adults than in children (*p* < 0.001, **Table 6**).

**Table 6:** Culture-independent P1 adhesin genotype results of clinical samples from adults and pediatric patients with CAP collected in Beijing in 2017.

P1 genotype	Adult	Children	Total	*X^2^*	*P*
**P1-1**	21 (56.76%)	124 (83.78%)	145 (78.38%)	12.76	< 0.001
**P1-2**	16 (43.24%)	24 (16.21%)	40 (21.62%)		
**Total**	37	148	185		

### 3.7 *M. pneumoniae* culture

In total, 375 *M. pneumoniae* strains were cultured from the clinical samples, including 361 strains from children and 14 strains from adults. None of the 23 samples that failed to be typed during the culture-independent P1 protein genotype assessment were cultured. The culture rate was significantly higher for P1-1 samples (321/605, 53.06%) than for P1-2 samples (54/192, 28.13%) (*p* < 0.01, **Table 7**).

**Table 7:** Positive rate of *M. pneumoniae* culture in strains with different P1 adhesin genotypes.

Culture Result	P1-1	P1-2	Failed to type	Total	*X^2^*	*P*
**Culture success**	321 (53.06%)	54 (28.13%)	0 (0.00%)	375 (45.73%)	56.45	< 0.01
**Culture fail**	284 (46.94%)	138 (71.88%)	23 (100%)	445 (54.27%)		
**Total**	605	192	23	820		

## 4 Discussion

*M. pneumoniae* is the most common cause of respiratory tract infection, accounting for 28% -40% of CAP cases in children (Waites et al., 2017; Shah, 2019; Meyer Sauteur et al., 2020b), and it is characterized by typical periodic epidemics. The relationship between the genotype of P1 adhesin and its variation with the periodic prevalence and severity of *M. pneumoniae* infection is attracting increasing attention. Understanding the P1 genotype of *M. pneumoniae* and its migration trend of subtypes may be useful for disease surveillance and control. Our study found that *M. pneumoniae-*infected patients in Beijing, Shenyang, and Baotou mainly carried the P1-1 genotype from 2017 to 2019, although the prevalence of the P1-2 genotype increased over time.

We detected *M. pneumoniae*, performed culture-independent P1 typing, and cultivated the microbe using 1,907 clinical samples collected from 2017 to 2019. The *M. pneumoniae* positivity rate was 43.00%, and the positivity rate was lower in adults than in children, highlighting the importance of *M. pneumoniae* as a cause of CAP in children, either inpatients or outpatients. For adults, we included only inpatient CAP patients, a study on outpatient CAM patients would strengthen our findings.

At present, five main methods are utilized to study the epidemiology and drug resistance mechanism of *M. pneumoniae*: multi-site variable number tandem repeat analysis (MLVA), multi-site sequence typing (MLST), single nucleotide polymorphism (SNP), variable number tandem repeat (VNTR), and P1 typing based on the repeat number of P1 protein-coding gene. Among these, VNTR and MLVA are commonly used in the genotyping of infectious diseases due to their greater discriminatory power (Yan et al., 2014).

In 2009, Degrange et al. (2009) screened VNTR in the gene sequence of *M. pneumoniae* using software and selected 5 species-specific VNTRs, named Mpn1, Mpn13, Mpn14, Mpn15, Mpn16. A total of 26 MLVA genotypes A-Z were identified according to the number of these five VNTR loci. However, Benitez et al. (2012) found that the Mpn-1 locus in the MLVA genotyping method was unstable. After 10 generations, 75% (6/8) of strains showed changes in the number of Mpn-1 repeats. Subsequently, researchers from various countries agreed that unstable Mpn1 locus should be excluded (Chalker et al., 2015), retaining only Mpn13, Mpn14, Mpn15, and Mpn16 to improve scoring stability. However, this change caused a significant reduction in the ability of MLVA typing to identify *M. pneumoniae* strains. Studies (Copete et al., 2018) showed that 4-5-7-2 of the improved MLVA genotypes were equivalent to the traditional P1 types, while 3-5-6-2 and 3-6-6-2 were equivalent to P1s. MLST is another widely used method for bacterial genotyping including for *M. pneumoniae*. Brown et al. (2015) developed an MLST scheme for *M. pneumoniae*, identifying 12 different sequence types (ST). SNP Genotyping is performed by analyzing DNA sequence polymorphisms caused by single nucleotide variations. However, MLST and SNP typing are mainly used in some studies due to cost and efficiency problems.

The PCR restriction fragment length polymorphism (PCR-RFLP) method requires the isolation and purification of *M. pneumoniae* strains from clinical samples. Because culturing *M. pneumoniae* is time consuming and not routinely performed in bacteriological practice, a culture-independent P1 typing method was established (Dumke et al., 2006). This method is more economical and efficient, as it directly identifies *M. pneumoniae* from clinical samples, making it widely used. Moreover, studies examining the relationship between macrolide resistance in M*. pneumoniae* and genotyping mainly use P1 genotyping and MLVA (Spuesens et al., 2010; Guo et al., 2019).

Adhesion is a key component of the pathogenic process of *M. pneumoniae*, and P1 adhesin is the most important adhesion protein of *M. pneumoniae* (Vizarraga et al., 2020), as it forms the tip structure in combination with P40/P90 (Vizarraga et al., 2020). P1 adhesin is involved in the immunopathogenic effects of *M. pneumoniae* (Fan et al., 2017), highlighting its role in infection by this microbe. The P1 genotype was established in the 1990s (Dallo et al., 1990), and P1 genotyping is the earliest and most mature *M. pneumoniae* genotyping method. Studies from different regions have reported the dominant genotype of P1 adhesin. For example, in 2011–2012, P1-2 was the predominant genotype in Slovenia (Kogoj et al., 2015) and South Africa (Carrim et al., 2018). However, P1-1 was dominant in the same period in Germany (Dumke et al., 2015), Sweden (Gullsby et al., 2019), France (Pereyre et al., 2013), Japan (Suzuki et al., 2017), China (Sun et al., 2017), and Thailand (Whistler et al., 2017), supporting the regional characteristics of the dominant P1 genotype.

A shift in the genotype of *M. pneumoniae* strains has been reported in several studies (Sasaki et al., 1996; Pereyre et al., 2007; Dumke et al., 2010). These data suggested the existence of periodic changes in the P1 genotype in *M. pneumoniae* even in the same region (Sasaki et al., 1996; Kenri et al., 2008). Considering that mycoplasma pneumoniae infection has the characteristics of periodic prevalence, it is speculated that P1 typing may be used in epidemiological studies (Atkinson et al., 2008). The relationship between the P1 genotype and the periodic epidemic of mycoplasma pneumoniae has gradually become a hot research topic. A study (Sasaki et al., 1996) in Japan conducted P1 typing of *M. pneumoniae* isolates from 1976 to 1995 and found that the periodic transformation of the P1 genotype may be related to the epidemic. Dumke et al. (2004) found that host pre-infection with a certain subtype would cause subtype-specific immunity, which had a strong impact on the type of bacteria that survived the secondary infection through animal experiments. This theory explains the phenomenon that subtype shifts in outbreaks of mycoplasma pneumoniae infection. In the current study, we used a culture-independent P1 typing method to investigate the P1 genotype in clinical samples collected from 2017 to 2019. Although P1-1 remained the dominant genotype, the proportion of P1-2 strains increased over time. Meanwhile, the proportion of P1-1 strains did not differ among Beijing, Shenyang, and Baotou, consistent with the results of Xiao et al (Yan et al., 2020). This trend was also similar to that observed by Jiang et al. (2021) during the same study period, but it contradicted the findings of a later study by Guo et al (Guo et al., 2022). The P1 genotype significantly differed between adults and children, similar to the findings of Yan et al (Yan et al., 2020). Interestingly, a study from Henan Province, China, found that by 2021, the proportion of P1-2 strains increased to 50% (Li et al., 2022), a trend consistent with our study in both period and region. A subsequent study from Shanghai, China, found that the proportion of P1-1 type increased significantly in the outbreak of *M. pneumoniae* in children after the COVID-19 pandemic (Zhu et al., 2024). From the epidemiological point of view, these findings suggest the importance of continuous monitoring of P1 genotype changes.

P1 adhesin is an important pathogenic gene of Mycoplasma pneumoniae, which can directly damage host bronchial epithelium (Miyata and Hamaguchi, 2016) and induce intense cellular and humoral immunity to participate in the pathogenic process of *M. pneumoniae* (Jacobs et al., 1990; Atkinson et al., 2008). After adhesion, *M. pneumoniae* caused disease by producing peroxide, Superoxide anion (Atkinson et al., 2008), and community-acquired respiratory distress syndrome toxin (CARDS TX) (Peters et al., 2011), as well as activating immune cells to produce inflammatory factors (Hoek et al., 2005). P1-1 may have higher reproductive capacity in patients with lower host immune responses. Furthermore, P1-1 genotype strains infection could induce a more severe respiratory immune response, worsen respiratory symptoms, and favor opportunistic infections (You et al., 2025). Based on the processes of pathogenesis and pathogenic of M. *pneumoniae*, the relationship between P1 genotype and disease severity has also been extensively studied. Unfortunately, the conclusions were inconsistent.

A study from Slovenia in 2014 reported that pediatric patients carrying P1-2 genotype strains tend to experience more severe disease compared to those infected with P1-1 strains and that patients infected by carrying P1-2 strains might be more prone to central nervous system and cardiovascular complications (Rodman Berlot et al., 2018; Rodman Berlot et al., 2021). In contrast, studies from Jiangsu Province (Xu et al., 2024) and Jilin Province (You et al., 2025) in China found that P1-1 genotype strain infection was associated with more severe clinical outcomes. Additionally, a study in Switzerland from 2016 to 2020 found that *M. pneumoniae* genotypes do not influence clinical outcomes (Meyer Sauteur et al., 2021). These differing results highlight the uncertainty in the relation between P1 genotype and disease severity. In our study, the P1 genotype did not differ between outpatients and inpatients or between children with severe pneumonia and those with general pneumonia. Our result aligns with the findings of Meyer et al (Meyer Sauteur et al., 2021), Nilsson et al. from Sweden (Nilsson et al., 2010), and several studies from Wuhan (Xu et al., 2024), Shanghai (Chen et al., 2024), Beijing (Jiang et al., 2024), and Henan (Li et al., 2022), China, but contrasts with those of studies in Slovenia (Rodman Berlot et al., 2018; Rodman Berlot et al., 2021). Further research is needed to clarify these discrepancies.

For strains cultured from 2017 to 2019, the culture positivity rate was higher for P1-1 strains than for P1-2 strains (53.06% vs. 28.13%). At present, there is no clear evidence to confirm this phenomenon, and we speculate that it may be related to the following factors. First, the sensitivity of different P1 genotype *M. pneumoniae* strains and the use of antibiotics before sampling may affect the positive rate of culture. Second, the self-synthesis and reproduction different P1 genotype strains may be different. All this speculation needs further evidence. The low positivity rate in *M. pneumoniae* culture and the time required for results confirm the advantages of the culture-independent P1 genotype method.

Some limitations of this study should be acknowledged. First, all samples in this study were collected from tertiary hospitals. In addition, the strains were mainly isolated in Beijing, and their classification in other regions is not well understood. Second, this study only investigated the P1 genotype, and different P1-1 and P1-2 variants could not be evaluated. These shortcomings will be addressed in future studies.

## 5 Conclusion

In conclusion, this study found that *M. pneumoniae* persisted as one of the dominant etiologic causes of CAP in children in China from 2017 to 2019. During the period, P1-1 remained the dominant genotype of *M. pneumoniae*, nonetheless the proportion of P1-2 strains increased over time. The P1 genotype was not associated with disease severity. However, continuous monitoring of P1 genotype trends remains important for mycoplasma pneumoniae control efforts.

## Data Availability

The original contributions presented in the study are included in the article/supplementary material. Further inquiries can be directed to the corresponding authors.
